# Bright Luminal Sign on High b-Value Diffusion-Weighted Magnetic Resonance Enterography Imaging as a New Biomarker to Predict Fibrotic Strictures in Crohn’s Disease Patients: A Retrospective Preliminary Study

**DOI:** 10.3390/jimaging10110283

**Published:** 2024-11-07

**Authors:** Luca Pio Stoppino, Stefano Piscone, Ottavia Quarta Colosso, Sara Saccone, Paola Milillo, Nicola Della Valle, Rodolfo Sacco, Alfonso Reginelli, Luca Macarini, Roberta Vinci

**Affiliations:** 1 Department of Medical & Surgical Sciences, Section of Diagnostic Imaging, University of Foggia, Viale Luigi Pinto n. 1, 71122 Foggia, Italy; luca.stoppino@unifg.it (L.P.S.); ottaviaquartacolosso@gmail.com (O.Q.C.); sara.saccone@unifg.it (S.S.); pmilillo@ospedaliriunitifoggia.it (P.M.); luca.macarini@unifg.it (L.M.); roberta.vinci@unifg.it (R.V.); 2 Department of Medical & Surgical Sciences, Section of Gastroenterology, University of Foggia, Viale Luigi Pinto n. 1, 71122 Foggia, Italy; nicola.dellavalle@gmail.com (N.D.V.); rodolfo.sacco@gmail.com (R.S.); 3 Department of Precision Medicine, University of Campania “L. Vanvitelli”, 80138 Naples, Italy; alfonso.reginelli@unicampania.it

**Keywords:** Crohn’s disease, MRE, magnetic resonance enterography, MR enterography, DWI, inflammatory bowel disease, fibrotic strictures, biomarker

## Abstract

A retrospective analysis was conducted to investigate how a bright luminal sign on high b-value diffusion-weighted imaging (DWI) could be considered as a new biomarker for identifying fibrotic strictures in Crohn’s disease (CD). Fibrotic strictures, due to excessive deposition of extracellular matrix following chronic inflammatory processes, can be difficult to distinguish from inflammatory strictures using endoscopy. This study was performed on 65 patients with CD who underwent MRE, and among them 32 patients showed the bright luminal sign on high b-value DWI. DWI findings were compared to pre- and post-contrast MRE data. Luminal bright sign performance results were calculated using a confusion matrix, the relationship between categorical variables was assessed by the χ2 test of independence, and the Kruskal–Wallis test (ANOVA) was used for the assessment of statistical significance of differences between groups. The results indicated a high sensitivity (90%) and specificity (85%) of the bright luminal sign for fibro-stenotic CD and a significant correlation between DWI luminal brightness and markers such as the homogeneous enhancement pattern (*p* < 0.001), increase in enhancement percentage from 70 s to 7 min after gadolinium injection (*p* < 0.001), and submucosal fat penetration (*p* = 0.05). These findings indicate that DWI hyperintensity can be considered as a good non-invasive indicator for the detection of severe intestinal fibrosis and may provide an efficient and accurate method for assessing fibrotic strictures. This new non-invasive biomarker could allow an early diagnosis of fibrotic stricture, delaying the onset of complications and subsequent surgery. Moreover, further evaluations through larger prospective trials with histopathological correlation are needed to confirm these results and completely determine the clinical benefits of DWI in treating CD.

## 1. Introduction

Crohn’s disease (CD) is a chronic relapsing inflammatory disease, which causes progressive and irreversible damage to the bowel. In CD, inflammation affects the entire intestine, with the distal ileum being the most affected area. A single triggering factor has not yet been identified, and it is reported in the literature that the pathogenesis of CD arises from the interplay of concurrent factors such as the environment, immune system disorders, susceptibility genes, and alterations in the host’s microbiome. Inflammatory cells are involved in sustaining active disease, resulting in disruption of the intestinal mucosa, and thus many therapies have as their target the suppression of inflammatory and pro-inflammatory cytokines [1].

The highest incidence of CD is seen in individuals aged 15 to 25, but it can affect people of all ages [2]. In a systematic review combining all epidemiological studies on IBD globally since 1990, it was found that the prevalence of CD is higher in Western nations compared to newly industrialized countries, particularly in North America and Western Europe, where it reaches an incidence ranging from 3 to 20 cases per 100,000. In countries undergoing industrialization in Asia and the Middle East, studies indicate a growing incidence of CD [3,4,5].

Since patients with CD commonly undergo alternating periods of flares and remissions, they may present with various non-specific symptoms that may delay an accurate diagnosis for several years.

Key symptoms of Crohn’s disease include fatigue, fever, abdominal pain, diarrhea, and weight loss (typically around 10–20%, resulting from reduced oral intake and malabsorption). Rectal bleeding is an uncommon symptom that can occur when the distal colon is affected by CD. The symptoms can be different every time the pathology is in the acute phase, and it is often misinterpreted as irritable bowel syndrome [2]. The average diagnostic delay ranges from 9 to 18 months from symptom onset, with longer delays correlating with increased risk of intestinal stenosis requiring surgery [6,7,8]. Isolated small bowel disease may manifest with diverse constitutional symptoms. Initially, around 20% of patients may present with complications such as intestinal strictures, abscesses, or fistulas. Additionally, common extraintestinal manifestations (EIMs) in CD comprise large joint arthritis, uveitis, iritis, episcleritis, erythema nodosum, and pyoderma gangrenosum [9].

As a result of chronic and severe inflammation, inflammatory mechanisms in CD could trigger excessive production of extracellular matrix (ECM) components [10]. The tissue repair process may involve a reduction in the lumen’s diameter, resulting in intestinal strictures and, eventually, obstruction. In this process, inflammation and fibrosis are closely interconnected mechanisms that coexist at various degrees within intestinal strictures [11].

According to the Montreal Classification [12], the course of the disease can be penetrating, stricturing, or non-stricturing/non-penetrating. However, this classification is not stationary and the clinical history of a patient may evolve or may show mixed types, such as the coexistence of fistulas and abscesses with strictures, conditions that increase the surgical risk [13]. Typically, at the time of diagnosis, it is identified as an inflammatory disease, but over the subsequent 10–15 years, it may evolve into a condition involving strictures, penetrating complications, and the development of fistulas and abscesses in nearly all patients. [14].

Up to 70% of patients with CD will need surgery during their lifetime due to the onset of strictures, which remain an indication for surgical intervention with or without penetrating complications [15]. Nevertheless, in CD strictures may not always be a permanent condition since they can present different degrees of inflammation and fibrosis. It is rare in CD that a stricture is exclusively fibrotic or inflammatory [16].

Considering that the management and treatment of inflammatory-based strictures are different from those for fibrotic ones, it is essential to determine which form is predominant. In particular, inflammatory strictures can be treated effectively with medical therapies, including corticosteroids or targeting TNFα, while fibrotic ones are a permanent condition that can only be managed through mechanical interventions such as endoscopic balloon dilation, strictureplasty, or surgical resection. Surgery remains the primary procedure for managing stenosis due to bowel fibrosis, yet post-operative recurrence often occurs, potentially resulting in the development of re-stenosing disease and requiring additional surgeries [16,17]. Hence, an accurate evaluation of intestinal fibrosis in CD is crucial for guiding clinical management.

The use of endoscopy is limited in the transmural evaluation of CD, but it presents the gold standard in the evaluation of mucosal healing. However, endoscopy is an invasive assessment and that limits its repeated use during disease monitoring. Moreover, up to 50% of CD patients diagnosed radiologically by cross-sectional imaging assessment have normal endoscopy findings [18,19].

As cross-sectional imaging modalities, entero-computed tomography (ETC) and intestinal ultrasound (IUS) show high efficacy. However, considering the close monitoring of CD patients, the use of ionizing radiation is not recommended. Meanwhile, with IUS, diagnosis and standardization of follow-up could be difficult, especially in inexperienced operators.

Among cross-sectional imaging techniques, magnetic resonance enterography (MRE) offers a transmural assessment of bowel loops (edema, wall thickening, enhancement post contrast) and can identify extra-intestinal disease and complications, features that make MRE particularly well-suited in CD patients [20]. In order to define the extent of fibrosis in CD strictures, Rimola et al. [21] introduced the concept of MRE gain of enhancement among the early and late phases after gadolinium-based contrast media injection. In fact, they found that the percentage of enhancement increase (>24% of enhancement gain) between 70 s and 7 min correlates with marked fibrosis.

The purpose of our study was to determine if DWI was able to identify marked intestinal fibrosis in CD patients using MRE post-contrast findings (homogeneous pattern of enhancement at 7 min and percentage of enhancement gain) as the reference standard.

Unfortunately, biomarkers, endoscopy, and histology cannot determine the extent of fibrosis within the strictures. Likewise, no imaging modalities have been validated to quantify fibrosis. However, emerging imaging techniques could be useful in the assessment of the fibrosis within the strictures.

## 2. Materials and Methods

### 2.1. Study Population

A retrospective study was performed on 65 patients with CD who underwent MRE in the period from December 2020 to May 2024. Among them, 32 CD patients (22 men and 10 women; mean age, 37.5 ± 7.2 years; range 18–56 years) received a final radiological diagnosis of fibro-stenosis and were included in this study. The baseline characteristics of the study population are presented in Table 1.

According to Rimola et al. [21], the radiological pattern of fibrosis is defined by the presence of a post-contrast homogeneous pattern of enhancement at 7 min and progression of enhancement between 70 s and 7 min. The percentage of gadolinium enhancement gain between 70 s and 7 min was calculated according to Formula (1). The exclusion criteria are given in Table 2.

Formula (1): percentage of gadolinium enhancement gain between 70 s and 7 min.

% Gain = [(WSI 7 min − WSI 70 s)/(WSI 70 s)] ∗ 100(1)

### 2.2. Imaging Parameters

MRE scans were acquired in the supine position with a 1.5 T scanner (Achieva dStream, Philips Medical System, Eindhoven, The Netherlands) equipped with a phased-array-16-elements coil (Sense XL Torso coil). All patients received 1000–1500 mL of iso-osmotic polyethylene glycol (PEG) solution orally one hour before their examination. This solution was prepared by dissolving a granular powder comprising PEG (58.32 g), sodium sulfate (5.69 g), sodium bicarbonate (1.69 g), sodium chloride (1.46 g), and potassium chloride (0.74 g) in water (Selg 1000, Promefarm, Milan, Italy). After 30 min of oral contrast administration, a coronal T2-weighted scan was performed to monitor an adequate intestinal distension.

T2-weighted images with and without fat saturation and DUAL Fast Field Echo (FFE) in the axial plane, as well as Balanced Fast Field Echo (BFFE) images in the coronal plane, were part of the acquisition protocol. Three-dimensional T1-weighted high-resolution isotropic volume excitation (THRIVE) with fat saturation sequences for dynamic contrast enhancement was obtained before and at 30–40 s, 70–90 s, and 120–140 s after intravenous administration of 0.1 mL/kg body weight of gadolinium chelate (Gd-BT-DO3A, Gadovist™, Bayer Healthcare, Leverkusen, Germany) at a rate of 1.0 mL/s. Subsequently, a DW single-shot spin-echo echo-planar sequence (SE-EPI) with a chemical shift selective fat suppression technique with b factors of 0, 300, 600, and 800 s/mm^2^ was performed. Finally, a 3D THRIVE sequence in the axial plane was acquired 420–450 s after contrast media injection. The parameters of the MRE protocol are shown in Table 3.

### 2.3. Image Analysis

For each fibro-stenotic intestinal segment, diffusion restriction was estimated both qualitatively and quantitatively. Qualitative assessment was characterized by the absence or presence (0/1) of DWI luminal hyperintensity in the pathological segment. For quantitative assessment, the average of apparent diffusion coefficient (ADC) values were obtained from three regions of interest, each with an area between 5 and 15 mm^2^ drawn both inside the lumen and on the intestinal wall. 

**Figure 1:**
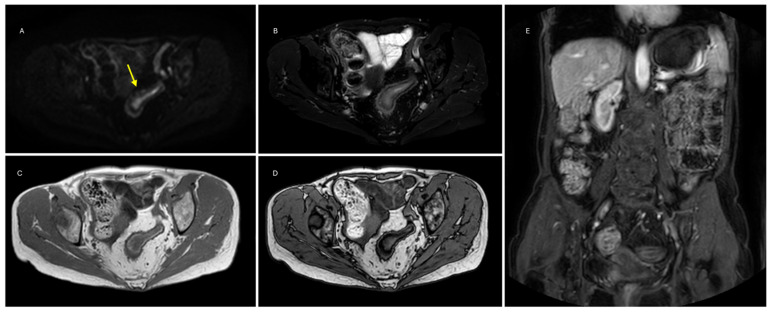
(**A**) DWI on high b-value image showing the bright luminal sign at the sigmoid tract (yellow arrow). (**B**) T2w fat-saturation image shows no signs of submucosal edema. (**C**,**D**) T1 IP-OOP showing diffuse submucosal adipose infiltrate. (**E**) Post-contrast image with layered pattern of enhancement at 70 s.

**Figure 2:**
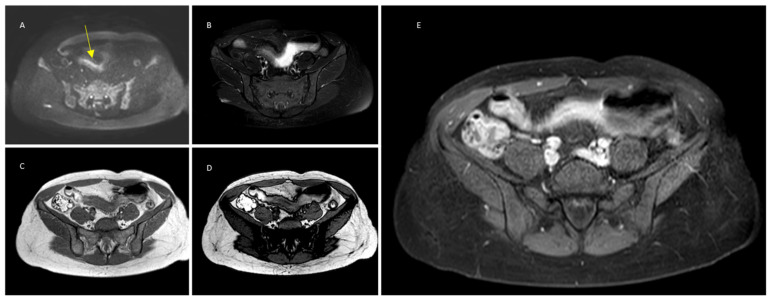
(**A**) DWI on high b-value image showing the bright luminal sign at the ileal tract (yellow arrow). (**B**) T2w fat-saturation image shows no signs of submucosal edema. (**C**,**D**) T1 IP-OOP showing diffuse submucosal adipose infiltrate. (**E**) Post-contrast image with homogeneous pattern of enhancement at 7 min.

The following MRE qualitative parameters were also recorded (Figure 1 and Figure 2): wall thickness, submucosal edema on T2, submucosal fat infiltration on both T2-fat sat and DUAL FFE, the presence of strictures (defined as a 50% reduction in the normal luminal diameter) and prestenotic dilatation, and post-contrast pattern of enhancement at 70 s (homogeneous, layered, or mucosal).

Two certified abdominal radiologists (reader 1 with 10 years of experience and reader 2 with 3 years of experience in MRE interpretation) performed qualitative and quantitative assessments of all MRE examinations.

### 2.4. Statistical Analysis

Luminal bright sign performance results were calculated using the initial sample of 65 patients (with and without strictures at radiological assessment), using patients with positivity for the sign as a reference standard. The results were defined in terms of accuracy, confusion matrix, sensitivity, specificity, positive predictive value (PPV), and negative predictive value (NPV).

Continuous variables were expressed as medians with interquartile ranges (IQRs). Absolute frequencies and percentages were used to describe categorical variables. The relationship between categorical variables was assessed by the χ2 test of independence. To examine the statistical significance of differences between groups, the Kruskal–Wallis test was used.

Interobserver agreement between the two observers was analyzed by using the intraclass correlation coefficients (ICCs) with a two-way model (<0.40, poor; 0.41–0.60, fair; 0.61–0.80, good; and 0.81–1.00, excellent correlation). All analyses were performed using GraphPad Prism version 8 for Mac OS X (GraphPad Software, San Diego, CA, USA, www.graphpad.com (accessed on 18 October 2024)).

## 3. Results

All patients with CD examined showed the presence of pathological segments with strictures, and among them, 29 showed the sign of luminal hyperintensity in DWI sequences with high-b values. Only three patients with fibro-stenotic pathology did not present the sign of luminal hyperintensity. In the group of patients with non-stenosing CD, only five of them showed the luminal bright sign (probably associated with peristalsis or meteorism).

Luminal bright sign performance in patients with fibro-stenotic CD is shown in Table 4.

As expected, the MRE parameters closely associated with DWI luminal hyperintensity were the presence of a homogeneous pattern of enhancement at 7 min (*p* < 0.001) and the percentage of gain of enhancement between 70 s and 7 min (*p* < 0.001). The DWI luminal hyperintensity also showed a significant correlation with submucosal fat infiltration and with luminal stenosis (*p* = 0.05). Bowel wall thickness and submucosal edema on T2w were not correlated with DWI luminal hyperintensity (*p* = 0.13 and 0.64, respectively; Table 5). 

For interobserver reliability, ICCs for all MRE measurements demonstrated a strong correlation between reader 1 and reader 2. There were perfect correlations for the percentage of gain enhancement and good to excellent correlations for the other parameters evaluated (Table 6).

## 4. Discussion

Contrary to other studies in the literature that have not found a utility of DWI sequences in the MRE protocol [22], our study focused on the usefulness of DWI and detected in this sequence the presence of a diagnostic biomarker for fibro-stenotic CD.

In our study, we demonstrated that the bright luminal sign obtained with the high b-value on DWI sequences has high performance in the diagnosis of fibro-stenotic CD. In addition, there is a significant correlation between DWI luminal brightness and other fibro-stenotic MRE findings (homogeneous enhancement pattern, submucosal fat penetration).

Several studies have shown the usefulness of DWI and ADC map in the evaluation of active-phase parietal inflammation [23,24], and that in combination with T2-weighted sequences, they can be used instead of contrast-enhanced MRI sequences in the detection of active inflammation in CD [25]. Furthermore, according to Foti et al. [26], the combination of conventional MRE sequences and DWI can define and provide a grading of intestinal parietal fibrosis.

Cross-sectional imaging modalities, such as computed tomography (CT), magnetic resonance imaging (MRI), and ultrasonography (US) are the most widespread non-invasive examinations for assessing both transmural and extramural lesions. Among these, MRE is radiation-free and highly accurate for the diagnosis of CD and evaluating its progression. It is frequently used throughout the disease’s progression to assess disease activity and luminal severity and to detect extra intestinal complications (abscesses, fistulas or strictures). On the contrary, CTE is not considered as the best tool in perianal fistula evaluation due to its poor accuracy in soft tissue evaluation. However, transperineal ultrasound is an effective diagnostic tool in assessing a possible inflammatory process of the perianal region [27].

Several studies have assessed the effectiveness of imaging in distinguishing between the fibrotic and inflammatory parts of strictures. However, only some of them directly compared these techniques to histology.

Histological analysis of surgical specimens remains the gold standard for diagnosing fibrosis. As reported by Chen et al. [28], the most important histological change in fibro-stenotic bowel strictures is smooth muscle hypertrophy and hyperplasia. CD, in addition to active and chronic inflammation and fibrosis, may present neuronal and adipose hyperplasia. Specifically, in CD strictures, normal submucosal collagen and adipose tissue are replaced by fibrotic tissue, combined with muscularis mucosa hyperplasia, referred to as the muscularization of the submucosa. The muscularis propria also becomes thickened and expanded due to collagen deposition [28]. All these factors concur to cause the thickening of the bowel wall seen on MRI and result in luminal narrowing. These are inversely correlated, because even a small improvement in the wall thickening is sufficient to produce a large increase in the luminal area [29].

DWI quantifies changes in water kinetics resulting from interactions among cell membranes, macromolecules, and tissue alterations that affect Brownian motion and fluid distribution, with its quantitative index being the ADC value [30,31]. In summary, increased freedom of water molecule movement leads to greater signal attenuation on diffusion-weighted imaging (DWI) compared to the T2-weighted sequence [32]. In the inflamed bowel wall, the extracellular space reduces due to heightened cell density and viscosity, lymphatic dilatation, and granulomatous formation. This leads to restricted water molecule diffusion, reflected in an elevated DWI signal intensity and a diminished ADC value [33,34]. Additionally, the accumulation of collagen in the bowel wall restricts the movement of water molecules [30]. Tielbeek et al. [35] and Kovanlikaya et al. [36] have reported that ADC values are inversely related to the degree of intestinal fibrosis and can distinguish fibrotic from non-fibrotic intestinal walls. In a previous study, it was shown that the accuracy of DWI in the assessment of intestinal fibrosis depends on the degree of wall inflammation coexisting; ADC can accurately distinguish fibrosis in mildly inflamed bowel walls, but not in moderate or severe inflammation, because a severe inflammatory condition can interfere with ADC assessment of fibrosis [37]. 

For our study, we recruited 65 CD patients, whereof 32 of them were candidates for surgery for fibro-stenotic disease.

Through this study, we observed that the luminal bright sign is closely associated with fibro-stenosating CD, achieving high percentages of accuracy (88%), sensitivity (90%), and specificity (85%).

Also, we confirmed that the percentage of contrast medium gain is not only very useful for studying parietal fibrosis in CD patients but also shows an excellent correlation with DWI luminal hyperintensity.

Moreover, the DWI luminal hyperintensity showed a close association with the homogeneous pattern of enhancement at 7 min and submucosal fat infiltration. As reported by Zappa et al. [38], we also found that the homogeneous pattern on the delayed phase is commonly associated with marked fibrosis. This can be attributed to the slower contrast diffusion in bowel segments with a high percentage of fibrotic tissue.

Our study had some limitations. First, the study was retrospective with a small patient cohort; in the future, larger randomized prospective trials are required to validate our findings.

Another limitation was the lack of histopathological analysis. Third, we only selected patients with marked fibrosis, excluding the subset of patients with a low degree of fibrosis or coexisting low degree of fibro-inflammation.

## 5. Conclusions

The strength of our work lies in the use of a new radiological sign such as DWI luminal hyperintensity with high accuracy, sensitivity, and specificity to recognize severe fibrosis in the intestinal wall. Our study has shown that DWI is a useful tool in the evaluation of CD, especially in distinguishing severe fibrotic stenosis.

In our opinion, the combination of conventional MRE findings (such as luminal narrowing, extension of the pathologic tract, and pre-stenotic dilatation) and the luminal bright sign on DWI sequences may provide a means of early detection of fibrotic bowel segments, contributing to better outcomes for patients affected by CD.

## Figures and Tables

**Table 1:** Demographic and clinical characteristics.

Characteristics	Number of Patients (*n*)
Patients	32
Male (%)	22 (69%)
Female (%)	10 (31%)
Age at diagnosis (median)	29.2
Disease duration (years, mean SD)	7.1 (2.8)
Fibro-stenotic localization	
-Rectum	0
-Sigmoid/Left colon	2
-Transverse colon	0
-Right colon	1
-Ileum	32
Therapy at any time of MRE	
-Anti-TNF (%)	14 (44%)
-Corticosteroids (%)	9 (28%)
-Immunosuppressant (%)	7 (22%)
-No treatment (%)	2 (6%)

**Table 2:** Exclusion criteria.

Exclusion Criteria
Age < 18 Radiological diagnosis of active inflammation Previous intestinal surgery
Presence of abdominal abscess or internal fistula No clinical and radiological evidence of fibro-stricturing CD

**Table 3:** MRE protocol.

	T2-TSE	T2-SPAIR	DUAL-FFE	BFFE	DYNAMIC THRIVE	DWI	THRIVE
Plane	axial	axial	axial	coronal	coronal (3D)	axial	axial (3D)
Slice thickness (mm)	5	5	5	5	1.5	6	3
FOV (mm)	450 × 450	400 × 400	450 × 450	400 × 400	420 × 420	450 × 450	420 × 420
TR (ms)	385	3100	140	2.9	5.5	3144	5.5
TE (ms)	80	80	4.6/2.3	1.4	0	89	0
Flip angle (degree)	90	90	80	40	10	90	10

**Table 4:** Bright luminal sign performance.

Diagnostic accuracy	88%
Sensitivity	90%
Specificity	85%
Positive predictive value (PPV)	85%
Negative predictive value (NPV)	90%

**Table 5:** DWI luminal hyperintensity with associated MRE findings. Bold values indicate those values with statistically significant differences. *p* ≤ 0.05 was considered statistically significant.

	Pathological Segment *Without* DWI Luminal Hyperintensity (*n* = 3)	Pathological Segment *With* DWI Luminal Hyperintensity (*n* = 29)	*p*-Value
Wall thickness (mm)	6.1 (5.45; 7.6)	9.2 (8.63; 11.1)	0.13 ^b^
Submucosal edema on T2w	3 (100%)	3 (10.3%)	0.64 ^a^
Submucosal fat infiltration on T2 fat sat or DUAL FFE	**1 (33.3%)**	**24 (82.75%)**	**0.05** ^a^
Luminal stenosis with prestenotic dilatation	**1 (33.3%)**	**25 (86.2%)**	**0.05** ^a^
Enhancement 7 min: - Homogeneous - Layered - Mucosal only	**0 (0%)** **1 (33.3%)** **1 (33.3%)**	**27 (93.1%)** **1 (3.4%)** **0 (0%)**	**<0.001** ^b^
Percentage of gain enhancement 70 s–7 min	**11 (9.4; 13.1)**	**32 (28.5; 39.7)**	**<0.001** ^b^

^a^ χ^2^ test. ^b^ Kruskal–Wallis test.

**Table 6:** Intraclass correlation coefficient (ICC) between two observers for all MRE parameters.

	ICC	*p*-Value
Wall thickness (mm)	0.868	0.001
Submucosal edema on T2w	0.854	<0.001
Submucosal fat infiltration on T2 fat sat or DUAL FFE	0.909	<0.001
Luminal stenosis with prestenotic dilatation	0.889	<0.001
Enhancement 7 min: - Homogeneous - Layered - Mucosal only	0.785 0.716 0.871	<0.001
Percentage of gain enhancement 70 s–7 min	0.958	<0.001

## Data Availability

The data presented in this study are available upon request from the corresponding author.
